# Transactions of the Pathological Society of Philadelphia

**Published:** 1840-02-22

**Authors:** 


### TRANSACTIONS OF THE PATHOLOGICAL SOCIETY OF PHILADELPHIA.

*February 3d*, 1840.

The President, Dr. Gerhard, in the Chair.

*Case of dislocation of the Cervical Vertebræ. Presented by Dr.* Randolph. *Reported and read by* J. F. Meigs, M. D.

M. R., a German, aged thirty-two, a strong, able-bodied man, was brought into the Pennsylvania Hospital on the 26th of January, 1840. At an early period of the evening before, while proceeding home in a state of intoxication, he, by some accident, fell backwards into an area of about seven feet in depth. Judging from the position in which he was lying when found, and from the existence of a small lacerated wound upon the crown of the head, it is probable that he fell directly upon the head, producing at the same time, extreme flexion of the cervical vertebræ, and a lateral twist from left to right. He was insensible when discovered about two hours afterwards, but was soon restored to consciousness by the use of frictions, &c.

When brought to the hospital, his intellect was clear, he answered questions, and complained chiefly of violent pain in the back of the neck. There was paralysis of the arms and legs; pulse slow and labouring; respiration abdominal; no headach, or, in fact, any cerebral symptom whatever. There was a lacerated wound of about an inch and a quarter in length, situated upon the top of the head, involving merely the integument. No contusion could be seen upon the back of the neck. Ordered four scarified cups to be applied to the neck, and ten grains of Dover's powder to be given.

*27th—Morning.* Has passed a bad night; very restless, complaining constantly of violent pain in the neck; no headach; intellect clear; pupils slightly contracted. Pulse fifty-six, of moderate volume, hard and tense. Respiration eighteen, diaphragmatic; the inspiration is full and quick, expiration rather slow. Skin warm and soft. Tongue dryish, covered with a light coat of whitish fur. Thirst very urgent. A pint and a quarter of urine drawn off by the catheter, of natural appearance; no stool since the accident. There is paralysis both of motion and sensation of the superior extremities, with the exception of the shoulders: of the trunk below the nipples, and of the inferior extremities. The action of the diaphragm remains perfect. In the parts not mentioned as paralysed, muscular power and sensation remain perfect. The loss of contractile power in the bladder is shown by the patient's entire inability to expel the urine when the catheter is introduced. Upon raising him into the sitting posture, the head falls heavily forward, greatly increasing the pain in the neck. No irregularity in the line of the vertebræ can be detected. Ordered V. S. ℥xvi.

℞. Hyd. Chl. Mit., et Pulv. Jalapæ $\overline{aa}$ gr. x., Chart. i., stat. sumenda.

*Evening.*—The only alteration is that the skin has become cool and soft. ℞. Pulv. Doveri gr.x.

28*th.*—Has not slept through the night; extremely restless; constantly demanding water; two stools, evacuations natural. Pulse sixty, moderately full and strong. Respiration as before. Skin of upper part of body and of arms warmer than natural, of inferior extremities cool. Tongue as before; no difficulty in protruding it. Paralysis remains the same. Catheter introduced twice a day. Mucilaginous diet.

29*th.*—Very restless through the night; complains principally of the pain in the neck. Pulse sixty-six, of considerable strength. Respiration more rapid than formerly, twenty-six. Tongue becoming dry and brownish; skin warm; no headach; intelligence good; no appetite; thirst urgent; one stool; paralysis as before; this morning, for the first and only time, observed slight erection of the penis. Urine is now becoming thick, whitish, and of an offensive odour. Ordered Tr. Opii gtts. lx. Died at 11 P. M.

*Autopsy thirty-six hours after death.*—Upon laying bare the upper part of the spinal column, there is found a complete luxation forwards of the fifth, upon the sixth cervical vertebræ. The posterior edges of the oblique processes of the fifth vertebræ are thrown in front of the anterior edges of those of the sixth; the ligaments uniting their spinous processes, and the corresponding yellow ligaments are entirely torn through, thus leaving a space of about half an inch between the bony bridges, in which the medulla spinalis, inclosed in its membranes, is completely exposed. The capsular ligaments surrounding the oblique processes are torn, leaving bare their articular cartilages. The intervertebral substance is separated from the inferior surface of the body of the fifth vertebra, so as to expose the bone without giving rise to any fracture. The anterior vertebral ligament is half torn through, the ruptured fibres being those situated upon the left side of the body. The muscular and ligamentous structure passing between the transverse processes of the two vertebræ, are much stretched and loosened, but not lacerated. The left transverse process is fractured at its base. There is a large quantity of black, coagulated blood, filling up the vertebral canal lying upon the outside of the dura mater. Dura mater natural, except that it has a slight red tinge from imbibition. Pia mater of a rose tint, from a number of minute vessels ramifying over it. Medulla, opposite to the seat of injury, converted into a soft, pulpy mass, of a dull white colour, with a slight admixture of red, from inflammation. This softening extends downwards as far as the first dorsal vertebra, though in less degree. In the head nothing abnormal was found, except great fulness of the posterior venous sinuses, probably the result of gravitation.

*Remarks.*—The dislocation in this case was evidently produced by the violent flexion of the neck, resulting from the whole weight of the body having been thrown upon the head in the fall. The fracture of the left transverse process, and the rupture of the fibres of the anterior vertebral ligament, found on the left side, were probably caused by the twist from left to right. It is rare that displacement to so great an extent takes place, without more considerable injury to the bony tissues than the insignificant one of fracture of the transverse process, which, it appears to us, can have but little effect in the production of luxation. That it does occur, however, even without any fracture whatever, is proved by three cases reported by Ollivier in his work on the Spinal Marrow. In two of these, the fifth cervical vertebræ were dislocated forwards upon the sixth, in one of which the violence was so great as to produce complete rupture of the spinal marrow, the ends of which were retracted above and below, for some distance, into the canal of the vertebræ. In the third case, a man having a sack of grain upon his back, bent far forwards to throw it off, and in so doing, the whole weight of the bag fell upon his head while thus strongly flexed; at this very moment he heard a crack, accompanied by a sudden and violent pain in the region of the neck, and fell down paralysed. He died thirty-six hours after the accident; and a luxation forwards of the sixth upon the seventh cervical vertebra, without any fracture, was discovered. The diagnosis, of course, was not difficult: the paralysis, the violent pain in the neck, the peculiar character of the respiration, the absence of all cerebral symptoms, pointed at once to the upper part of the vertebral column as the seat of injury.

### THE MEDICAL EXAMINER.

PHILADELPHIA, FEBRUARY 22, 1840.

